# Efficacy and safety of once-weekly semaglutide for the treatment of type 2 diabetes: Protocol for a systematic review and meta-analysis

**DOI:** 10.1097/MD.0000000000010420

**Published:** 2018-04-20

**Authors:** Fang-Hong Shi, Hao Li, Min Cui, Zai-Li Zhang, Zhi-Chun Gu, Xiao-Yan Liu

**Affiliations:** a Department of Pharmacy, Renji Hospital; b Department of Pharmacy, Shanghai Children's Medical Center, School of Medicine, Shanghai Jiaotong University, Shanghai, China.

**Keywords:** glucagon-like peptide-1 receptor agonists, meta-analysis, semaglutide, type 2 diabetes mellitus

## Abstract

**Background:**

It is a great challenge for type 2 diabetes mellitus (T2DM) patients to maintain optimal glycemia, control body weight, blood pressure, and avoiding hypoglycemia. Glucagon-like peptide-1 (GLP-1) receptor agonists (GLP-1 RAs) can stimulate glucose-dependent insulin while inhibit glucagon secretion, delay gastric emptying, reduce appetite, and energy intake. Recently, a new once-weekly GLP-1 RAs, semaglutide, has been registered to treat patients with T2DM.

**Methods:**

We will search Medline, Embase, Cochrane Library, and the ClinicalTrials.gov Website up to February 2018. Studies will be screened by title, abstract, and full text independently in duplicate. Phase III randomized controlled trials (RCTs) reports efficacy and safety data of semaglutide will be eligible for inclusion. Outcome variables will be assessed included glycemic control indexes (glycosylated hemoglobin [HbA1c]%, fasting plasma glucose [FPG], self-monitoring of blood glucose [SMPG], postprandial self-monitoring of blood glucose [PSMPG]), blood pressure indexes (systolic blood pressure [SBP], diastolic blood pressure [DBP], and pulse rate), body weight control indexes (body weight, body mass index [BMI], and waist circumference), and any adverse events (including adverse events [AEs] varying degrees and AEs occurring in ≥5% patients by preferred term or other of clinical interest). Assessment of risk of bias and data synthesis will be performed using STATA software (version 12, Statacorp, College Station, Texas). Outcomes will report by weight mean difference (WMD) and risk ratios (RRs) and their 95% confidence intervals (95% CIs). Heterogeneity among studies will be evaluated using the *I*^2^ statistic.

**Results:**

This review will evaluate glycemic, blood pressure, body weight control, and any adverse events of semaglutide as compared with other therapies.

**Conclusion:**

Our study will provide a comprehensive picture of semaglutide in T2DM.

## 1 Introduction

From 1999 to 2014, the prevalence of type 2 diabetes mellitus (T2DM) has increased from 8.8% to 11.7%.^[1]^ Most of T2DM patients are confined to abdominal obesity patients with ages over 45.^[1]^ There is a tremendous challenge for T2DM patients to achieve glycated hemoglobin (HbA1c) targets. For individualized hypoglycemic options, balancing weight and avoiding the occurrence of hypoglycemia are important considerations.^[2,3]^ Weight loss is also considered important in T2DM patients with overweight, as it is known to improve glycemia control and reduce cardiovascular risk factors.^[4]^ Glucagon-like peptide-1 (GLP-1) receptor agonists (RAs) are a reasonable choice for T2DM treatment.^[3]^ GLP-1 RAs can stimulate insulin secretion in a glucose dependent way and inhibit glucagon, resulting in a significant reductions of glycemia and lower risk of hypoglycemia compared with other hypoglycemic agents.^[5]^ As for the treatment of T2DM by first generation of GLP-1 RAs, once daily or twice daily administration is unavoidable. Recently, scientists make efforts on developing GLP-1 RAs for once weekly administration, which could improve patients’ adherence, leading to a better effectiveness compared with the first generation of GLP-1 RAs.^[6,7]^ Semaglutide (Novo Nordisk, Denmark) is a new GLP-1 RAs with 94% structural homology to native GLP-1, similar in structure with liraglutide, but less susceptible to degradation by enzyme protease dipeptidyl peptidase-4 (DPP-4) and more enzymatically stable.^[8]^ Semaglutide has been approved by the US Food and Drug Administration on December 5, 2017, as an adjunct to diet and exercise to improve glycemia control.^[9]^ Although semaglutide has been evaluated or is being evaluated in some large-scale, long-term randomized trials. A comprehensive evaluation of efficacy and safety of semaglutide are still needed. In this study, we will conduct a systematic review and meta-analysis to present an overview of the efficacy and safety of semaglutide in patients with T2DM.

## 2 Methods

This review will perform follow the principle of Preferred Reporting Items for Systematic Reviews and Meta-Analyses (PRISMA) guidelines and conduct following a priori established protocol (PROSPERO: CRD42018084958).^[10]^ Ethical approval is not required because this is a literature-based study.

### 2.1 Literature search strategy and study selection

**Figure 1:**
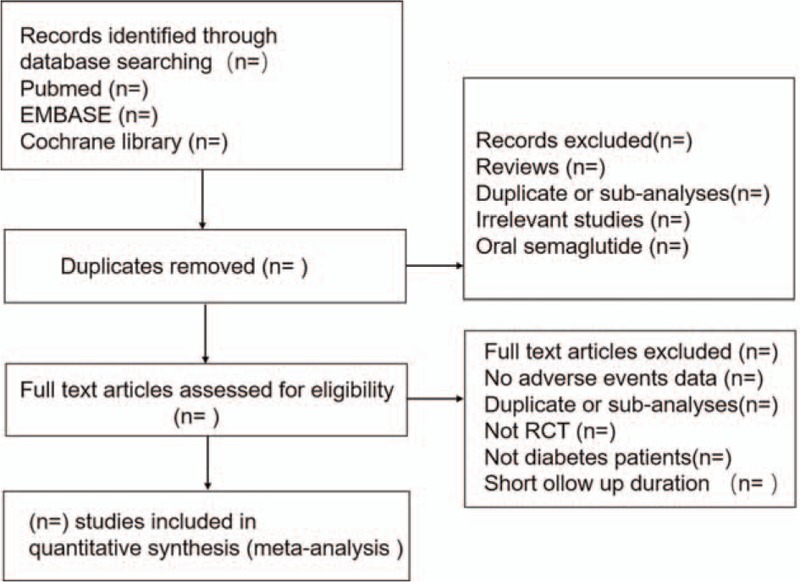
Flow chart of the search process.

We will perform a comprehensive literature search of relevant databases including Medline, Embase, Cochrane Library, and the ClinicalTrials.gov Website up to February 2018. The search strategy will be enacted according to the guidance offered from the Cochrane Handbook with the following Medical Subject Heading (MeSH) terms and variants: “type 2 diabetes” or “type 2 diabetes mellitus,” “Semaglutide” or “NN9936” or “NN9934” or “NN9935” or “ozempic” and “clinical trial” or “controlled clinical trial” or “randomized controlled trial,” and any possible spellings of “semaglutide” and “diabetes”, (Table 1). The search strategy is listed in Table 1, and others will be optimized according to the requirement. All the studies will be selected and confirmed by FHS and HL and any disagreements are resolved by consensus or by consulting a third author (ZCG). Literatures that are not conformed to the inclusion criteria will be excluded. If the integrality of article is incomplete, we will email to the corresponding or first authors. The details of the selection process are shown in Fig. 1.

**Table 1:**
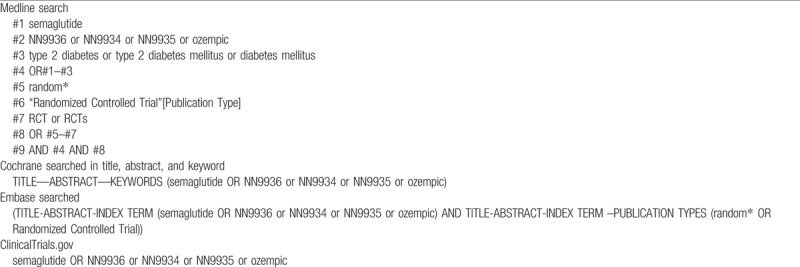
Electronic search strategies.

### 2.2 Outcome measures

This protocol proposes to assess the efficacy and safety of semaglutide. For the topic of glycemia control, we plan to evaluate glycosylated hemoglobin (HbA1c)%, fasting plasma glucose (FPG), self-monitoring of blood glucose (SMPG), postprandial self-monitoring of blood glucose (PSMPG). For the topic of body weight control, we plan to assess body weight, BMI, and waist circumference. As for blood pressure, SBP, DBP, and pulse rate will be analyzed. With respect to safety, we will assess any adverse events including AEs varying degrees and AEs occurring in ≥5% patients by preferred term or other of clinical interest.

### 2.3 Data extraction

The substantial contents of each selected articles will be extracted by FHS and HL, respectively. The information should be including these items: the name of the first author, NCT number, publication time, randomization and control therapies, sex, age, baseline HbA1c, durations of diabetes, renal function, and other details such as different controls, durations of follow ups, different dosages, and any efficacy and safety data. Any disagreements are resolved by consensus or by consulting a third author (ZCG).

### 2.4 Quality assessment

The Cochrane tool (Statacorp, College Station, Texas) will be used to assess the bias risks of studies,^[11]^ the following 7 items related to the bias risk will be assessed by FHS and HL: random sequence generation, allocation concealment, blinding of participants and personnel, blinding of outcome assessment, incomplete outcome data, selective reporting and other bias. Furthermore, these items should be judged by low risk, high risk, or unclear risk of bias. The disagreement will be settled by discussion or consult to the third author.

### 2.5 Data synthesis

We will use STATA12.0 to deal with the data from primary and second studies. Weight mean difference (WMD) will be analyzed and 95% confidence intervals (CI) presented in continuous variable and risk ratio (RRs) for dichotomous variable. We will use *I*^2^ statistic and *χ*^2^ test to assess heterogeneity in the studies. The heterogeneity will assess as follows: <50% are considered low heterogeneity, between 50% and 70% are considered moderate heterogeneity, while >70% are considered high heterogeneity. When *I*^2^ is >50%, a random effects model and DerSimonian–Laird method will be used to calculate the effect estimates whereas *I*^2^ <50%, a fixed effects model with the Mantel–Haenszel method are used. Furthermore, if quantitative synthesis is not appropriate, we will adopt qualitative description to assess the data.

### 2.6 Subgroup analysis

We will conduct subgroup analysis based on different dosages of semaglutide, durations of follow ups, and controls.

### 2.7 Sensitivity analysis

Sensitivity analysis will be conducted to identify the robustness of the result by omitting each of the study or excluding low-quality trials.

### 2.8 Reporting biases

Potential reporting biases will be performed by funnel plots. We will also perform Begg test and Egger test if asymmetry is showed by a visual inspection. *P* > .05 in Begg test and Egger test are considered no significant publication bias.

### 2.9 Ethics and dissemination

We aim to explore current evidence connected with the effectiveness and safety of once weekly semaglutide for the treatment of T2DM patients. The main outcome includes glycemia control indexes (HbA1c%, FPG, SMPG, PSMPG), body weight control indexes (body weight, BMI, waist circumference), blood pressure control (SBP, DBP, and pulse rate). This systematic review does not require ethical assessment because only indirect literature will be included and evaluated. Furthermore, the result will be disseminated as a literature review in related journal.

## 3 Discussion

T2DM is a chronic, progressive disease characterized mainly by persistent hyperglycemia. GLP-1 RAs can reduce body weight, which are particularly beneficial for T2DM patients accompanied with obesity.^[12]^ Semaglutide is generally used as an adjunctive to diet and exercise treatment in T2DM. Previous studies reported a great reduction of HbA1c% and body weight in patients with T2DM who were treated with semaglutide when compared with other therapies.^[13]^

Semaglutide showed a significant improve in controlling glycemia and bodyweight as compared with other GLP-1 RAs. Recent head-to-head studies indicate that semaglutide is superior to other once weekly administrated GLP-1 RAs (exenatide extended release and dulaglutide) with not just glycemia control but body weight control and other efficacy data.^[12,14]^ Whether semaglutide is more effective and less adverse effects is still elucidated. Furthermore, up to now, there is no relevant systematic review has been reported.

The purpose of this review is to assess the effect and safety of semaglutide in T2DM patients. In particular, we will identify the influence of efficacy and safety in different dosages of semaglutide, different controls, and follow up durations. Overall, we will give a comprehensive picture of semaglutide in efficacy and safety. In order to ensure the accuracy and reliability of the results, different authors will screen articles at least 3 times respectively. We intend to use sufficient evidence to guarantee credibility for this meta-analysis. Herein, this systematic review will be the first to evaluate the efficacy and safety of semaglutide in patients with T2DM, which may offer an objective and comprehensive understanding of semaglutide.

## Author contributions

FHS and HL exacted and analyzed the data and wrote the first draft of the protocol and MC helped with the design of the protocol. ZLZ submitted the registration on PROSPERO. ZCG revised the manuscript. XYL is the guarantors for the publication and take the responsibility for the paper. All authors participated in read and approved the final manuscript.

**Conceptualization:** Fang-Hong Shi, Hao Li, Zhichun Gu, Xiao-Yan Liu.

**Data curation:** Min Cui, Zai-Li Zhang.
